# Effectiveness of Endophytic and Rhizospheric Bacteria from *Moringa* spp. in Controlling *Pythium aphanidermatum* Damping-Off of Cabbage

**DOI:** 10.3390/plants12030668

**Published:** 2023-02-02

**Authors:** Buthaina Aamir Ali Al-Rahbi, Abdullah Mohammed Al-Sadi, Majida Mohammed Ali Al-Harrasi, Jamal Nasser Al-Sabahi, Issa Hashil Al-Mahmooli, Daniel Blackburn, Rethinasamy Velazhahan

**Affiliations:** 1 Department of Plant Sciences, College of Agricultural and Marine Sciences, Sultan Qaboos University, Al-Khoud, Muscat 123, Oman; 2 Central Instrumentation Laboratory, College of Agricultural and Marine Sciences, Sultan Qaboos University, Al-Khoud, Muscat 123, Oman; 3 Department of Soil, Water and Agricultural Engineering, College of Agricultural and Marine Sciences, Sultan Qaboos University, Al-Khoud, Muscat 123, Oman

**Keywords:** *Moringa olifera*, *Moringa perigreina*, *Brassica oleracea*, bacterial antagonists, biological control

## Abstract

In this study, endophytic and rhizospheric bacteria were isolated from *Moringa olifera* and *M. perigreina* from Oman, and their in vitro antagonistic activity against *Pythium aphanidermatum* was tested using a dual culture assay. The promising strains were tested further for their compatibility and potential for plant growth promotion, biofilm formation, antifungal volatile organic compound (VOC) production, and the biological control of *P. aphanidermatum* damping-off of cabbage (*Brassica oleracea* L.) under greenhouse conditions. A total of 12 endophytic and 27 rhizospheric bacteria were isolated from *Moringa* spp. Among them, *Bacillus pumilus* MPE1 showed the maximum antagonistic activity against *P. aphanidermatum* in the dual culture assay, followed by *Paenibacillus glucanolyticus* MPE3 and *Pseudomonas indica* MOR3 and MOR8. These bacterial isolates induced abundant morphological abnormalities in the hyphae of *P. aphanidermatum*, as observed via scanning electron microscopy. The in vitro cross-streak assay showed that these bacterial isolates were compatible among themselves, except for *P. indica* MOR8 × *P. glucanolyticus* MPE3. These antagonists released VOCs that restricted the growth of *P. aphanidermatum* in an in vitro assay. These antagonistic bacteria released 2,4-dimethylheptane and 4-methyloctane as the predominant volatile compounds. Of the four antagonistic bacterial strains, *P. indica* MOR8 was capable of forming biofilm, which is considered a trait that improves the efficacy of rhizosphere inoculants. The results of the greenhouse experiments showed that the soil treatment with *B. pumilus* MPE1 showed the highest reduction (59%) in the incidence of *P. aphanidermatum* damping-off in cabbage, evidencing its potential as a biological control agent for the management of this disease. Further research is needed to characterize the antifungal traits and activities of *B. pumilus* MPE1 and to assert its potential use against other soil-borne plant pathogens.

## 1. Introduction

Cabbage (*Brassica oleracea* L.), which belongs to the Cruciferae family, is one of the most important vegetable crops in the world. This crop suffers from a variety of bacterial, viral, fungal, and oomycete diseases [1]. Damping-off is one of the serious diseases affecting cabbage production worldwide. The pathogenic infection causes the pre-emergence mortality of seedlings. Various phytopathogens, including *Rhizoctonia solani* [2,3,4], *Pythium ultimum* [5], *Pythium aphanidermatum* [6], and *Fusarium moniliforme* [7] have been reported to cause damping-off in cabbage. Synthetic chemical fungicides are routinely used to control the damping-off disease of vegetable crops [8]. Due to environmental and food safety concerns, there is considerable interest in finding alternative methods to synthetic chemical pesticides. There are a few environmentally friendly alternatives for the control of damping-off in vegetable crops, such as soil solarization, topsoil replacement, and biofumigation [9,10]. One of the most promising and feasible methods may be biological control through the bioaugmentation of naturally occurring antagonistic soil microorganisms [11].

Both Cruciferae and Moringaceae plants, such as rapeseed, cabbage, cauliflower, broccoli, Brussels sprouts, turnip, Arabidopsis, radish, and moringa, are known to contain high quantities of glucosinolates (sulfur-containing glucosides) in their tissues, which upon hydrolysis by means of myrosinase, an endogenous enzyme, produce several breakdown products, including isothiocyanates (ITCs) and indoles [12,13,14]. The antifungal, antibacterial, and nematicidal activities of ITCs have been demonstrated [15,16]. Dhingra et al. [16] reported that soil drenching with allyl isothiocyanate (AITC) resulted in over 90% control of the *Rhizoctonia solani* damping-off of cabbage seedlings. The inactivation of intracellular enzymes has been reported as the mode of action of ITCs against bacteria [17].

ITCs are known to exude from the host roots [18] and interfere with the survivability of plant-beneficial rhizobacteria, including biological control agents (BCAs). ITCs obtained from rapeseed glucosinolates have been reported to disrupt the microbial community composition in the soil [19]. Chen et al. [20] showed that the endophytic fungal communities in cruciferous crops are influenced by the host plant species, types of tissues, and season. In an effort to isolate beneficial soil microorganisms, we isolated 13 morphologically distinct bacterial isolates from the rhizosphere soil of cabbage collected from commercial fields in Barka, Muscat (Oman). However, none of them showed an inhibitory effect against *P. aphanidermatum* (unpublished data), leading to our search for alternative environments from which these BCAs could be isolated. We hypothesized that among the culturable bacteria dwelling in the rhizosphere or inside the plant tissues, such as the endophytes of *Moringa* species, some would act as BCAs for Cruciferae species against soil-borne fungal phytopathogens. This hypothesis is based on the assumption that the antagonistic microorganisms obtained from glucosinolate-containing plants would be adapted to other glucosinolate-rich environments, such as the rhizosphere of Cruciferae species. The present work aimed to isolate endophytic and rhizospheric bacteria from *Moringa oleifera* and *M. peregrina* and then investigate their potential use in controlling the damping-off of cabbage caused by *P. aphanidermatum*.

## 2. Results

### 2.1. Screening and Identification of Bacteria with Antagonistic Activity against P. aphanidermatum

**Figure 1:**
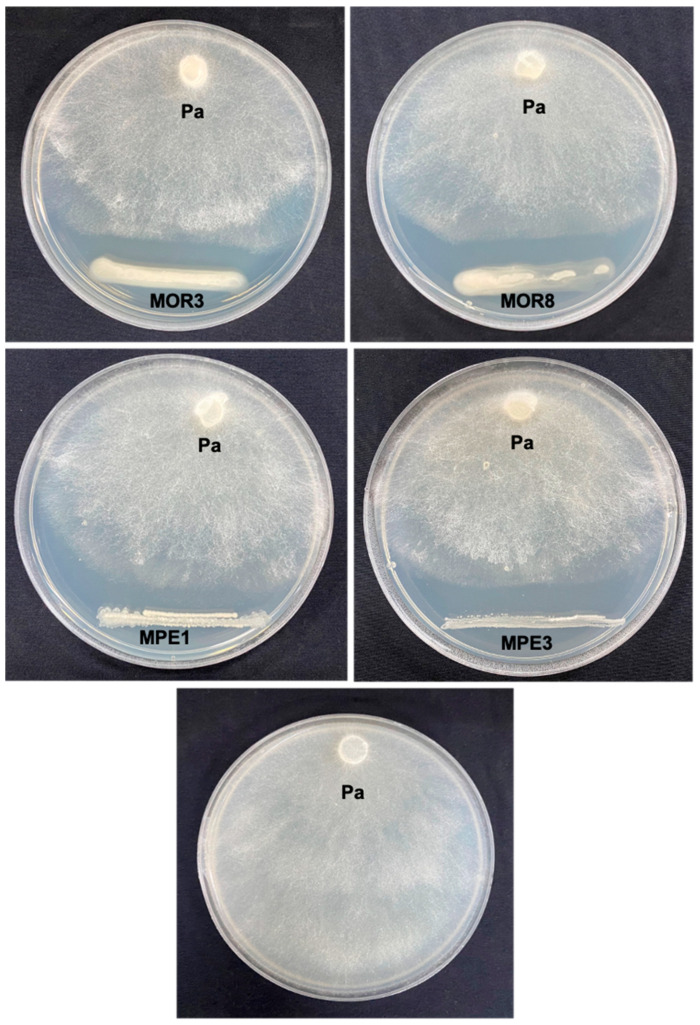
In vitro inhibitory effects of the rhizospheric and endophytic bacterial isolates from *Moringa* spp. on *Pythium aphanidermatum* (Pa).

A total of 39 morphologically different bacterial colonies (12 endophytic bacteria and 27 rhizospheric bacteria) were isolated from the roots and rhizosphere soil of *M. oleifera* and *M. peregrina* using NA medium. The antagonistic activity of these bacterial isolates toward *P. aphanidermatum* was tested in vitro using a dual culture method. Among them, 4 bacterial isolates (2 endophytic and 2 rhizospheric bacteria) designated MPE1, MPE3, MOR3, and MOR8 showed high levels of anti-oomycete activity (above 30%) and recorded 62.5%, 40.0%, 31.2%, and 31.2% mycelial growth inhibition, respectively, compared to the control (Table 1; Figure 1).

### 2.2. Molecular Identification of Bacterial Isolates

For the identification of the 4 selected antagonistic bacterial strains, an approximately 1400 bp 16S rRNA gene fragment was PCR-amplified and sequenced using the universal primer pairs 27F and 1429R. The 16S rRNA sequences of these 4 bacterial isolates were compared to the GenBank database using the BLASTn tool and identified as *Bacillus pumilus* MPE1 (98.8% identity), *Paenibacillus glucanolyticus* MPE3 (100.0% identity), *Pseudomonas indica* MOR3 (100.0% identity), and *Pseudomonas indica* MOR8 (100.0% identity). The 16S rRNA sequences of these bacterial isolates have been deposited in the GenBank under accession numbers ON908584, ON908543, ON928357, and ON928355 (Table 2).

### 2.3. Scanning Electron Microscopic (SEM) Observations of P. aphanidermatum Hyphae at the Inhibition Zone

**Figure 2:**
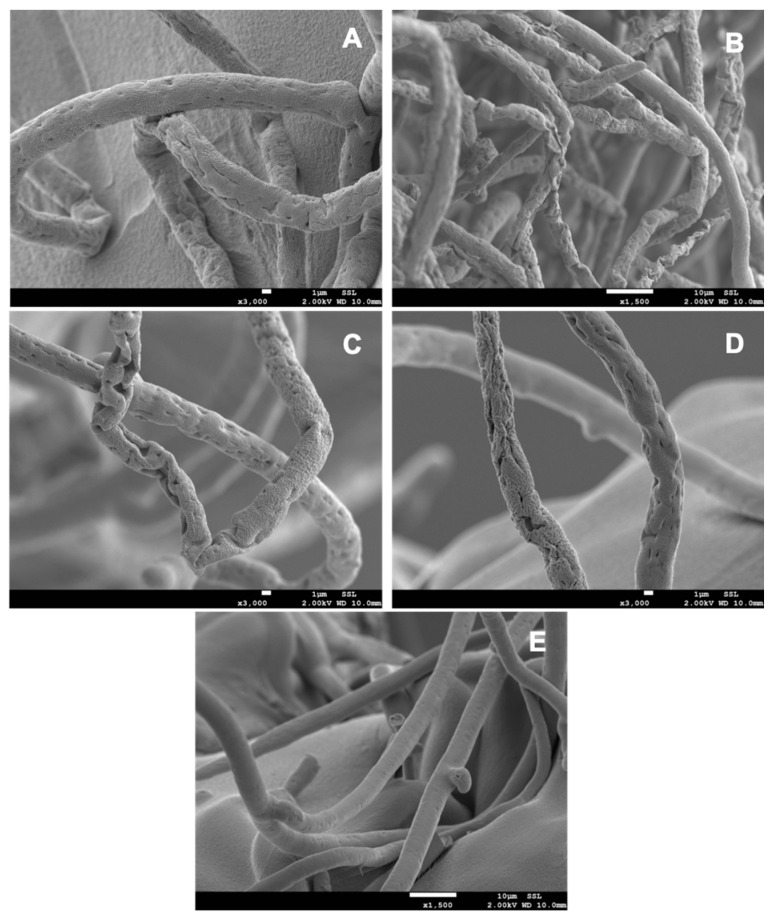
Scanning electron microscopic images showing the morphological abnormalities in the hyphae of *Pythium aphanidermatum* when co-cultivated with the antagonistic endophytic and rhizosphere bacteria from *Moringa* spp. (**A**) *P. aphanidermatum* co-cultivated with *P. indica* MOR3; (**B**) *P. aphanidermatum* co-cultivated with *P. indica* MOR8; (**C**) *P. aphanidermatum* co-cultivated with *B. pumilus* MPE1; (**D**) *P. aphanidermatum* co-cultivated with *P. glucanolyticus* MPE3; and (**E**) control.

The images from the scanning electron microscopic examination of the *P. aphanidermatum* hyphae at the zone of inhibition showed that the diffusible metabolites released by the antagonists caused abundant morphological changes in the mycelium of *P. aphanidermatum*. The shrinkage, pit formation, disintegration, and rough outer surface of the hyphae were commonly observed in *P. aphanidermatum* when co-cultivated with the antagonists (Figure 2). The mycelium of *P. aphanidermatum* grown in the absence of antagonists (control) was healthy, with a smooth outer surface.

### 2.4. Inhibitory Activity of Volatile Organic Compounds (VOCs) Produced by the Antagonistic bacterial Strains against P. aphanidermatum

The four selected antagonistic bacterial strains were then subjected to an in vitro sealed base plates assay. The results indicated that there was a reduction in the mycelial growth of *P. aphanidermatum* when paired with base plates containing the antagonistic bacterial strains compared to the control. The VOCs produced by *P. indica* MOR3 significantly suppressed the growth of *P. aphanidermatum* with 28.4% inhibition (Table 3). The VOCs of the other three selected bacterial strains *P. glucanolyticus* MPE3, *P. indica* MOR8, and *B. pumilus* MPE1 slightly suppressed the mycelial growth of *P. aphanidermatum* with 22.2%, 12.3%, and 12.3% growth inhibition, respectively; however, these results were not statistically significant (*p* < 0.05).

### 2.5. Chemical Analysis of VOCs Produced by the Antagonistic Bacterial Strains

The analysis of the VOCs released by *P. indica* MOR3, *P. indica* MOR8, *B. pumilus* MPE1, and *P. glucanolyticus* MPE3 by means of GC-MS revealed the production of 13, 16, 20, and 18 compounds, respectively (Table 4, Table 5, Table 6 and Table 7). Heptane, 2,4-dimethyl- (38.41%), octane, 4-methyl- (17.79%), and 3-Ethyl-3-methylheptane (13.36%) were the major VOCs produced by *P. indica* MOR3. Heptane, 2,4-dimethyl- (31.78%), octane, 4-methyl- (19.71%), and 3-Ethyl-3-methylheptane (14.63%) were the VOCs most produced by *P. indica* MOR8. *Bacillus pumilus* MPE1 produced carbamic acid, monoammonium salt (19.29%), heptane, 2,4-dimethyl- (18.03%), octane, 4-methyl- (9.78%), and 1,4-Pentadiene (8.03%) as the major compounds. The most abundant VOCs produced by *P. glucanolyticus* MPE3 were heptane, 2,4-dimethyl- (20.87%), octane, 4-methyl- (15.49%), and nonane, 5-(2-methylpropyl)- (14.37%).

### 2.6. Biofilm-Forming Ability of Antagonistic Bacterial Strains

A microplate assay was used to determine the biofilm-forming ability of the endophytic and rhizospheric antagonistic bacterial strains isolated from *Moringa* spp. Among the 4 bacterial strains tested, *P. indica* MOR8 was the only strain capable of significantly forming (*p* < 0.05) biofilm in vitro, as indicated by a higher OD value (A_600_ = 1.367) compared to the control (A_600_ = 0.134) (Table 8).

### 2.7. Plant Growth-Promoting Activities of Antagonistic Bacterial Strains

The in vitro inoculation of cabbage seeds with the selected antagonistic bacteria had no significant effect (*p* < 0.05) on the germination percentage, root length, shoot length, and seedling vigor after two weeks of growth (Table 9).

### 2.8. Compatibility of Antagonistic Bacterial Strains

**Figure 3:**
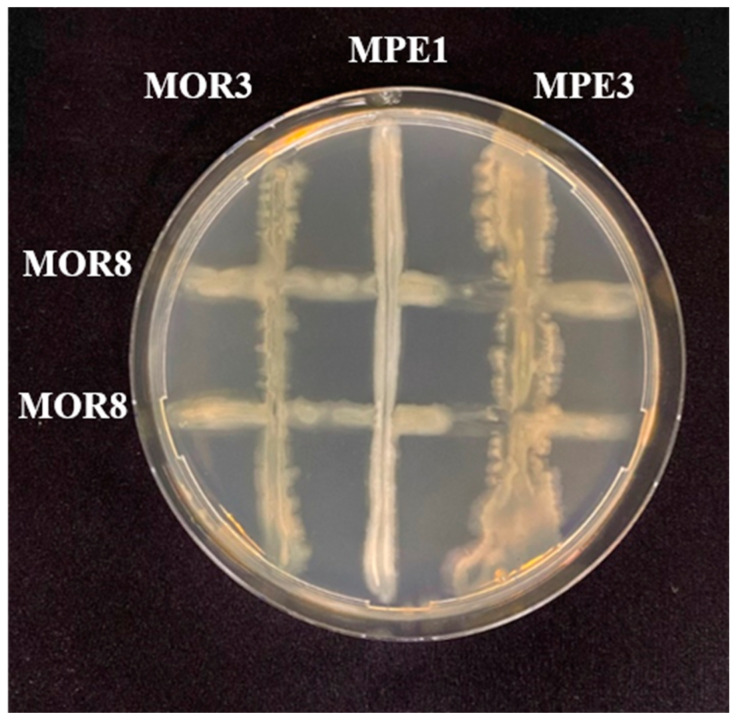
In vitro cross-streak assay showing the incompatibility between *Pseudomonas indica* MOR8 and *Paenibacillus glucanolyticus* MPE3. MOR3, *Pseudomonas indica*; MOR8, *Pseudomonas indica*; MPE1, *Bacillus pumilus*; MPE3, *Paenibacillus glucanolyticus*.

The compatibility of the bacterial strains was assayed in vitro using cross-streak assays in order to determine their possible future co-inoculation. All the bacterial strains, except for *P. indica* MOR8 and *P. glucanolyticus* MPE3, were compatible while growing on a nutrient agar medium. A clear zone of inhibition was observed only between *P. indica* MOR8 and *P. glucanolyticus* MPE3 (Figure 3).

### 2.9. Efficacy of the Bacterial Strains in the Biological Control of P. aphanidermatum Damping-Off of Cabbage

**Figure 4:**
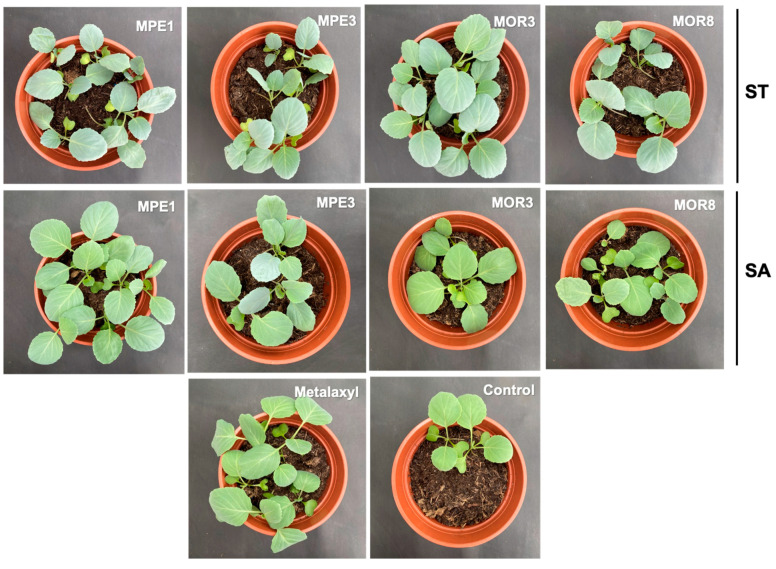
Effect of seed treatment (ST) or soil application (SA) with endophytic and rhizospheric bacteria from *Moringa* spp. on the incidence of *Pythium aphanidermatum* damping-off in cabbage.

A greenhouse pot trial using sterile soil was conducted to assess the efficacy of the antagonistic endophytic and rhizospheric bacterial strains isolated from *Moringa* spp. in the control of phytopathogenic *P. aphanidermatum* causing the damping-off of cabbage. The pathogen-infected controls recorded the highest damping-off disease incidence of 73.3% (Table 10). The seed inoculation with the BCAs was ineffective, and only the soil application of *B. pumilus* MPE1 significantly reduced the incidence of the damping-off of cabbage (59%) over the untreated control (Figure 4). The soil application of metalaxyl (0.1%) was used as a positive control treatment representing the standard pesticide treatment. It resulted in only a 45% disease reduction; however, these results were not statistically significant (*p* < 0.05). The other BCAs, namely *P. indica* MOR3, *P. indica* MOR8, and *P. glucanolyticus* MPE3, had no significant (*p* < 0.05) effect on reducing the damping-off disease incidence.

## 3. Discussion

The successful use of endophytic fungi and bacteria as well as plant growth-promoting rhizobacteria (PGPR) in plant disease management has been widely reported [21,22,23,24,25,26,27,28]. In this study, two bacterial strains, *Pseudomonas indica* MOR3 and *Pseudomonas indica* MOR8, isolated from the *Moringa olifera* rhizosphere and two endophytic bacterial strains, *Bacillus pumilus* MPE1 and *Paenibacillus glucanolyticus* MPE3, isolated from *Moringa perigreina* root tissues showing an inhibitory effect on the growth of *P. aphanidermatum* were isolated. Of the four bacterial strains tested, *B. pumilus* MPE1 exhibited the maximum antagonistic activity against *P. aphanidermatum* in a dual culture assay, followed by *P. glucanolyticus* MPE3, and *P. indica* MOR3, and *P. indica* MOR8.

The observations of *P. aphanidermatum* from the inhibition zone by means of SEM revealed diverse morphological abnormalities in the hyphae, including a rough outer surface, distortion, shrinkage, and pit formation due to the antagonistic effect of the bacterial isolates, while the hyphae of the pathogen grown in the absence of the antagonists were normal and had a smooth outer wall surface. Similar findings have been reported in *P. aphanidermatum* mycelium due to the antagonistic effect of *Pseudomonas resinovorans* [27], *Pseudomonas aeruginosa* and *Bacillus cereus* [29], and *Aspergillus terreus* [30]. The shrinkage of the *P. aphanidermatum* hyphae in the inhibition zone might be due to the leakage of the internal cell contents due to the activity of the diffusible antifungal metabolites of the antagonistic bacteria [31]. The observed in vitro antagonistic effect of the bacterial strains isolated from *Moringa* spp. on *P. aphanidermatum* in the dual culture experiment might be due to the diffusible antifungal compounds produced by the bacterial strains in the agar medium [32], while the variations in their inhibitory effects might be due to the quantity of production and toxicity of these compounds. These results suggest the production of antifungal metabolites to be one of the mechanisms of action of these bacterial strains on *P. aphanidermatum*.

Many PGPR and endophytes are known to produce antimicrobial VOCs that suppress the growth of phyopathogenic fungi [6,33,34,35]. Some VOCs are known to cause fungal cell membrane damage that results in the leakage of the intracellular contents and, finally, cell death [33,36,37]. Furthermore, microbial VOCs can spread over long distances and inhibit the growth of phytopathogens without direct contact and induce resistance mechanisms in plants [38,39,40]. Huang et al. [6] demonstrated that the antagonistic strains of *Bacillus mycoides* isolated from the tomato rhizosphere produced two VOCs, dimethyl disulphide (DMDS) and ammonia, which caused hyphal deformities in *P. aphanidermatum* and *Rhizoctonia solani*, the damping-off pathogens of cabbage. The in vitro two sealed base plates assay revealed that the VOCs of all four bacterial strains tested suppressed the mycelial growth of *P. aphanidermatum.* Among them, the VOCs of *P. indica* MOR3 exhibited the most potent inhibitory activity against *P. aphanidermatum*. The SPME-GC-MS profiling of the VOCs of these bacterial strains revealed the presence of 2,4-dimethylheptane (alkane) and 4-methyloctane (hydrocarbon) as the principal compounds in common between the bacterial strains. 2,4-dimethylheptane has been reported as a VOC in *Ficus tikoua* [41] and fungi [42,43]. The production of 2,4-dimethylheptane by *Fusarium verticillioides* has also been documented [44]. Jia et al. [45] demonstrated that the exogenous application of jasmonic acid enhanced the 4-methyloctane content in grapefruit and induced resistance against *Botrytis cinerea*. However, their antimicrobial role remains to be elucidated.

Among the four selected bacterial strains isolated from *Moringa* spp., *P. indica* MOR8 alone was capable of forming biofilm in vitro. The biofilm refers to a community of microbial cells embedded in a self-produced extracellular polymeric matrix (EPS) that adhere to a live or inert surface [46,47]. The formation of biofilm is recognized as a useful adaptive strategy of many microbial biocontrol agents to environmental conditions [48]. The biofilm increases the resistance of bacterial strains to various environmental stresses and allows them to effectively colonize the rhizosphere of plants [49]. Several microbial biocontrol agents are capable of forming biofilm, and this trait is generally assumed to help the strain colonization of the rhizosphere and their persistence [50,51,52,53]. In this study, no relationship was seen between the biofilm forming ability and biocontrol activity of the bacterial strains isolated from *Moringa* spp. However, Bais et al. [50] reported that *Bacillus subtilis* strain 6501, which is an efficient biocontrol agent against *Pseudomonas syringae* pv. *tomato*, formed a robust biofilm, whereas the ineffective biocontrol agent *B. subtilis* strain M1 did not form a biofilm.

Many antagonistic rhizobacteria are also considered plant growth-promoting rhizobacteria (PGPR) becuase they promote plant growth [54]. However, a few rhizobacteria are reported to suppress the growth of plants. For instance, the treatment of tomato seeds with *Klebsiella oxytoca* strain D1/3 isolated from the rhizosphere of tomato resulted in a reduction in the shoot length, root length, and vigor of the tomato seedlings [21]. It is evident from this study that the inoculation of cabbage seeds with the selected bacterial strains had no significant effect on the seed germination percentage, seedling root length, shoot length, and seedling vigor of the cabbage. These results indicate that there was no detrimental effect of any of the used bacterial strains on the cabbage seedlings’ growth.

To enhance the biocontrol efficiency, mixtures of microbial strains with different modes of action are desirable in the biocontrol of plant diseases [22,55]. The in vitro cross-streak assay in this study showed that, except for *P. indica* MOR8 and *P. glucanolyticus* MPE3, all the bacterial strain combinations were compatible between themselves. Dunne et al. [56] found that mixtures of *Stenotrophomonas maltophilia* strain W81 (capable of producing lytic enzymes) and *Pseudomonas fluorescens* strain F113 (capable of producing antibiotic 2,4-diacetylphloroglucinol) were more effective in controlling the damping-off of sugar beet compared to individual applications of either bacterial strain. Similarly, the combined application of *Bacillus pumilus* and *Rhizophagus intraradices* offered better protection for the common bean from *Rhizoctonia solani* root rot [57]. Ankati et al. [58] found that mixtures of *Streptomyces griseus* strains CAI-24, CAI-121, and CAI-127 + *Streptomyces africanus* KAI-32 + *S. coelicolor* KAI-90, or mixtures of *Streptomyces griseus* strain CAI-127 and *Streptomyces africanus* KAI-32, effectively controlled the chickpea wilt caused by *Fusarium oxysporum* f. sp. *ciceri.* In a similar study, a consortium of antagonistic bacterial strains consisting of *Exiguobacterium indicum* D1/8 and *Bacillus cereus* D1/17 were found to be effective in the control of the *P. aphanidermatum* damping-off of tomato compared to individual antagonists. Similarly, the combined application of *Pseudomonas aeruginosa* (bacterial biocontrol agent) and *Trichoderma harzianum* (fungal biocontrol agent) significantly reduced the severity of the *Fusarium* wilt of banana [59]. The compatibility between the antagonistic bacterial strains isolated from *Moringa* spp. in this study suggests their suitability to be tested as a consortium of bacterial inoculants in future studies investing robust treatment and effective disease control.

The results of the greenhouse experiments showed that the soil application with *B. pumilus* MPE1 was the most effective treatment against the damping-off of cabbage. However, the same bacterial strain when applied through seeds did not show a significant level of damping-off control. This might be due to the inability of these strains to form large populations from a relatively small inocula size, leading to differences in the population densities of the biocontrol agents between these forms of BCA application. The other bacterial strains had no significant (*p* > 0.05) effect on the damping-off disease incidence when applied either to seed or soil. Several species of *Bacillus*, including *B. subtilis* [60], *B. velezensis* [61,62], *B. amyloliquefaciens* [60,63], and *B. pumilus* [52] have been widely used as biocontrol agents in the control of diseases in crops. *Bacillus* spp. suppress plant pathogens by producing various metabolites, such as antibiotics, siderophores, lipopolysaccharides, and lytic enzymes, and by enhancing the expression of the defense mechanisms of plants [64]. *Bacillus* spp. are capable of producing endospores, which provide resistance to a wide variety of environmental stresses and make these bacteria suitable candidates for developing different formulations for the biocontrol of plant diseases. *B. pumilus* has been reported to be an antagonistic bacterium against *Fusarium oxysporum* f. sp. *lycopersici* causing the wilt of tomato [52]. This study is the first report of a *B. pumilus* strain that is capable of controlling the *P. aphanidermatum* damping-off of cabbage.

In conclusion, the endophytic bacterial strain *B. pumilus* MPE1 isolated from *M. perigreina* was able to inhibit the growth of *P. aphanidermatum* in vitro and significantly reduced the incidence of the damping-off of cabbage under greenhouse conditions when applied directly to the soil. This strain showed compatibility with the other isolated bacterial strains, and it had no adverse effect on cabbage growth or germination. Hence, this strain may be a potential biocontrol agent for the control of the *P. aphanidermatum* damping-off of cabbage, especially under organic cultivation. Further studies are needed to evaluate the performance of *B. pumilus* MPE1 under field conditions. In addition, studies are required to assess the biosafety, mode of action, and endophytic movement of this strain in cabbage plants. The tolerance mechanisms of *B. pumilus* MPE1 against glucosinolates should be studied. The role of 2,4-dimethylheptane and 4-methyloctane, the major VOCs produced by *B. pumilus* MPE1, in the suppression of *P. aphanidermatum* should also be determined.

## 4. Materials and Methods

### 4.1. Sample Collection

Root samples from six-month-old *Moringa peregrina* seedlings grown in pots in a shaded house as well as well-established *M. peregrina* and *M. oleifera* trees were collected from the Botanical Garden (23.5893° N, 58.1660° E) of Sultan Qaboos University (Oman) and transferred to the laboratory. The soil adhering to the roots (rhizosphere soil) was collected in sterile sandwich bags. The root samples and rhizosphere soil were used for the isolation of the endophytic and rhizospheric bacteria, respectively.

### 4.2. Isolation of Endophytic and Rhizospheric Bacteria from Moringa spp.

To isolate the endophytic bacteria, the root samples were washed thoroughly in running tap water and then in distilled water to remove any adhering soil particles. The roots were surface-sterilized by immersing them in 70% ethanol for 1 min, followed by immersing in 1% NaOCl for 5 min. After rinsing thoroughly in sterile distilled water (SDW) 3 times, the sterilized root tissues (500 mg) were ground with 500 µL of SDW using a mortar and pestle. An aliquot of the sap (100 µL) was spread evenly on nutrient agar (NA; Oxoid Ltd., Basingstoke, UK) plates (90 mm in diameter). After 2 days of incubation at 30 °C, the growth of the bacteria on the plates was checked and the bacterial colonies with varying morphological features, such as color, shape, margin, elevation, and texture, were selected and a pure culture was obtained via the streak-plate method [65]. To verify the effectiveness of the sterilization process, aliquots of the SDW used in the final rinse were also plated on NA medium. Three replications were made for each sample.

To isolate the rhizosphere bacteria, the rhizosphere soil (1 g) was mixed with 9 mL of SDW in 15 mL sterile polypropylene centrifuge tubes (Corning; Fisher Scientific, Loughborough, UK) and shaken vigorously for 1 min. Serial dilutions of the soil suspension were made with SDW, and 100 µL of the soil suspension (10^−4^, 10^−5^ and 10^−6^) was plated on NA medium [66]. Two replications for each concentration were prepared, and the plates were incubated at 30 °C. After two days, the bacterial colony growth on the NA medium was checked and pure cultures of the individual colonies were obtained as described above.

### 4.3. Isolation of Pathogen

*P. aphanidermatum* was isolated from the damping-off infected cabbage seedling collected from Barka, Al-Battinah, Oman using a potato dextrose agar (PDA) culture medium (Oxoid Ltd., Basingstoke, UK). The pure culture of the oomycete pathogen was obtained using the single hyphal-tip method [66], maintained on PDA slants, and stored at 4 °C. The identity of the pathogen was confirmed based on the morphological features and an analysis of the sequences of the rRNA gene internal transcribed spacer (ITS) regions, as described by Al-Sadi et al. [67]. The nucleotide sequence has been deposited in the GenBank (https://www.ncbi.nlm.nih.gov/genbank/) under accession number OQ253408 (accessed on 14 January 2023).

To test the pathogenicity of the isolate, 4 mycelial discs (6 mm dia) taken from a 3-day-old culture of *P. aphanidermatum* on PDA were transferred to sterile plastic pots (14 cm diameter and 10 cm depth) containing 400 g of sterilized potting mixture (Bulrush Horticulture Ltd., Ireland, UK) under aseptic conditions. Cabbage seeds (Agri-Cross F1; Agrimax Group S.L.U, Barcelona, Spain) were sown in the inoculated pots at a rate of 5 seeds/pot. The pots were incubated in a greenhouse at 27 °C with a 16 h photoperiod. The development of symptoms on the plants was recorded 10–15 days after sowing. To prove Koch’s postulates, the pathogen was re-isolated from the plants showing the symptoms of damping-off and confirmed via an analysis of the rDNA ITS region.

### 4.4. Antagonistic Potential of Bacterial Isolates against P. aphanidermatum

The in vitro dual culture technique was employed to test the antagonistic activity of the endophytic and rhizospheric bacterial isolates against *P. aphanidermatum* [21]. In a 9 cm diameter Petri dish containing ~20 mL of NA, 1-day-old bacterial culture was streaked at 1 cm apart from the edge of the Petri dish. On the opposite side, a 6 mm disc of 3-day-old *P. aphanidermatum* culture was placed 1 cm away from the edge of the Petri dish. The Petri dishes were sealed individually with parafilm and incubated at 27 °C for 72 h. Three replications were prepared for each bacterium. The Petri dish containing the NA medium inoculated with *P. aphanidermatum* only served as the control. The growth of *P. aphanidermatum* (from the center of the disc to the edge of the mycelium) as well as the inhibition zone (the distance between the bacterial isolates and the edge of *P. aphanidermatum* mycelium) were recorded. The bacterial isolates that showed high levels of inhibitory activity against *P. aphanidermatum* (above 30% inhibition) were selected for further studies.

### 4.5. Scanning Electron Microscopy (SEM) of P. aphanidermatum Mycelia

To observe the morphological changes in the *P. aphanidermatum* hyphae due to the antagonistic activity of the bacterial isolates, a small piece (0.5 cm) of the agar media containing the mycelium of *P. aphanidermatum* taken from the outer edge in the inhibition zone was cut, transferred to a small vial containing 1 mL of Karnovsky’s fixative buffer, and placed on a rotator mixer for 1 h. The fixative buffer was then removed and 1 mL of 0.1 M sodium cacodylate washing buffer, pH 7.4, was added and incubated at 25 °C for 10 min. The washing buffer was removed and 1 mL of washing buffer was added again and incubated at 25 °C for 10 min. Then, 1 mL of 2% osmium tetroxide in distilled water was added to each vial and the vials were placed on a rotator mixer for 1 h at 25 °C. Next, the vials were washed with distilled water 2 times each for 10 min, followed by washing with 25% ethanol for 10 min, 75% ethanol for 10 min, 95% ethanol for 10 min, and absolute ethanol 2 times each for 10 min. Subsequently, 1 mL of 50% hexamethyldisilazane diluted in ethanol was added to the vials and incubated for 30 min at 25 °C. Finally, 1 mL of 100% hexamethyldisilazane was added to the vials and incubated for 5 min. The vials were kept open overnight to dry and then fixed on stubs. The samples were visualized using a scanning electron microscope (JSM-7800F; JEOL USA, Inc., Peabody, MA, USA).

### 4.6. Molecular Characterization of Bacterial Isolates

#### 4.6.1. DNA Extraction

The bacterial isolates that showed promising antagonistic activity against *P. aphanidermatum* were selected and DNA was extracted from the overnight bacterial cultures using a Foodproof StarPrep Two kit (BIOTECON Diagnostics, Potsdam, Germany) according to the manufacturer’s protocol. The concentration and purity of the DNA were determined using a NanoDrop 2000 Spectrophotometer (Thermo Scientific, Waltham, MA, USA).

#### 4.6.2. Bacteria Strain Identification via 16S rRNA Gene Sequencing

The amplification of the 16S rRNA gene of the bacterial isolates was performed using the extracted DNA and two universal primers: 27F (5′-AGAGTTTGATCMTGGCTCAG-3′) and 1429R (5′-TACG GYTACCTTACGACTT-3′) [68]. In a 0.2 mL PCR tube, a PuReTaq Ready-To-Go PCR bead (GE Healthcare, Buckinghamshire, UK), 21 µL of sterile deionized water, 1 μL (20 pmol µL^−1^) of each primer, and 2 μL of DNA (50 ng µL^−1^) were added and thoroughly mixed. The PCR was carried out using a Veriti 96-well thermal cycler (Applied Biosystems, Singapore) with the following conditions: initial denaturation at 95 °C for 2 min, 35 cycles of denaturation (95 °C for 30 s), annealing (54 °C for 30 s), extension (72 °C for 1 min), and a final extension at 72 °C for 10 min. Finally, the PCR amplification was confirmed by running 5 µL of the PCR product (DNA) in 1% agarose (Thermo Scientific, Waltham, MA, USA) gel in Tris-borate-EDTA buffer (pH 8.0). The gel was viewed under a UV light using a Gene Flash (Syngene Bioimaging) system. The PCR products of the correct sizes were sequenced at Macrogen, Seoul, Republic of Korea. The obtained sequences were compared with the reference sequences available in the GenBank database of the National Institute of Health (NIH) using the BLAST program (http://www.ncbi.nlm.nih.gov/BLAST/) (accessed on 2 July 2022).

### 4.7. Production of Anti-Oomycete Volatile Organic Compounds (VOCs) by Bacterial Strains against P. aphanidermatum

The inhibitory activity of the VOCs released by the bacterial isolates against *P. aphanidermatum* was analyzed in two sealed base plates assays [40]. Briefly, a 6 mm mycelial disc of *P. aphanidermatum* taken from an active culture was placed in the center of a PDA medium (⅕ strength) in a 9 cm diameter Petri dish. The test bacterial suspension (50 µL; 10^6^ CFU ml^−1^) was transferred to the NA medium in a Petri dish (9 cm dia) and spread uniformly using a sterile glass spreader. The lids were removed, and both the base plates were paired and wrapped with two layers of parafilm. The culture plates were then incubated at room temperature (25 ± 2 °C). After 48 h of incubation, the diameter of the *P. aphanidermatum* mycelium was measured. The PDA Petri dish inoculated with the mycelial disc of *P. aphanidermatum* and paired with the non-inoculated NA Petri dish served as the control. Each treatment had three replicates, and the experiment was repeated twice.

### 4.8. Chemical Analysis of VOCs Produced by the Bacterial Isolates

First, 100 µL of overnight bacterial culture (10^6^ CFU ml^−1^) was transferred to 20 mL of sterile nutrient broth in a sterile GC-MS vial. The vials were sealed with parafilm to avoid the escape of volatile compounds and then placed in an incubator shaker for 48 h at 30 °C and 170 rpm. The vial was pierced with an SPME (Solid Phase Micro Extraction) device that absorbs VOCs from the headspace through fibers on the needle and secured with the router clamp for 40 min. Then, the SPME was placed on the GC-MS for 7 min and 34 s, and then removed. The GC-MS was carried out using a Shimadzu GC-2010 Plus gas chromatography system equipped with an Rtx-5MS 30 m × 0.25 mm ID; 0.25 μm GC capillary column coupled to a GCMS-QP2010 ULTRA MS [69]. The analysis of the compounds was completed within 30 min. The identification of the compounds was accomplished using the NIST 2011 v.2.3 and Wiley 9th edition mass spectrum libraries.

### 4.9. Biofilm-Forming Potential of Bacterial Strains

The ability of the bacterial isolates to form biofilm was assessed using the method described by O’Toole [70], albeit with slight modifications. A loopful of each bacterium was inoculated into 200 mL of nutrient broth (NB; HiMedia Laboratories, Mumbai, India) in a 500 mL conical flask. The inoculated flask was incubated overnight at 30 °C and 170 rpm. The optical density (OD) of the cells was adjusted to 0.2 at 600 nm. Then, 10 mL of the bacterial suspension was centrifuged at 3000 rpm and 4 °C for 5 min. The supernatant was discarded and the pellet was re-suspended in 10 mL of fresh NB. In a 96-well polystyrene plate, 200 µL of bacterial suspension was added and the plate was incubated at 30 °C. Ten replications were made for each bacterium, and the un-inoculated NB served as the control. After incubation for 24 h, the plate was washed with SDW. Then, 200 µL of crystal violet solution (0.1% *w*/*v* in distilled water) was added to the well and incubated for 15 min at room temperature (25 ± 2°C). The plate was washed with SDW five times and air-dried. Later on, 200 µL of 95% ethanol (Sigma-Aldrich, St. Louis, MO, USA) was added to the wells and incubated at room temperature for 30 min. At the end of the incubation, the absorbance of the solution was measured using a microplate reader (Multiscan Go; Thermo Fisher Scientific, Vantaa, Finland) at a wavelength of 600 nm.

### 4.10. Assessment of Plant Growth-Promoting Activities of Bacterial Strains

Each bacterium was grown in a 500 mL conical flask containing 200 mL of NB on an incubator shaker for 24 h at 30 °C and 170 rpm. The OD of the cells was adjusted to 1.0 at 600 nm. Then, 10 mL of the bacterial suspension was centrifuged at 3000 rpm and 4 °C for 10 min. The supernatant was discarded and the pellet was re-suspended in 10 mL of SDW. The cabbage seeds (Agri-Cross F1) were dipped in the bacterial suspension for 3 h at room temperature (25 ± 2 °C). The seeds that were soaked in SDW served as the control. Later on, 15 seeds were spread on a seed germination paper in a line at equal distance (2 cm) and covered with a germination paper strip. The germination paper, along with the seeds, was rolled and covered with a polyethylene sheet and secured with rubber bands. The rolls were then placed in a beaker containing 100 mL of distilled water and kept at room temperature (25 ± 2 °C). After 14 days of incubation, the root and shoot lengths of each seedling and the germination percentage were documented to calculate the vigor index (vigor index= (mean root length + mean shoot length) × (% germination) [71]. Three replications were maintained for each treatment.

### 4.11. Compatibility Analysis between Bacterial Strains

The compatibility between the bacterial strains was analyzed using the cross-streak assay method, as described by Al-Hussini et al. [21]. On an NA plate, a bacterial strain was streaked in two lines parallel to each other, and the other three test bacterial strains were streaked perpendicularly to the first bacterium. The plates were sealed with parafilm and incubated for 48 h at 30 °C. After incubation, the merger of the bacterial colonies at the intersection was observed and photographed. Three replications were prepared for each bacterium.

### 4.12. Evaluation of the Efficacy of the Bacterial Strains in the Biological Control of P. aphanidermatum Damping-Off of Cabbage

The bacterial strains *Pseudomonas indica* MOR3, *Pseudomonas indica* MOR8, *Bacillus pumilus* MPE1, and *Paenibacillus glucanolyticus* MPE3, which showed high antagonistic effect on *P. aphanidermatum* under in vitro conditions, were selected to evaluate their efficacy under greenhouse conditions in the control of the *P. aphanidermatum* damping-off of cabbage. The sterile plastic pots (14 cm diameter and 10 cm depth) were filled with 400 g of sterilized potting soil (Bulrush Horticulture Ltd., Ireland, UK). The starter cultures of the bacterial isolates were prepared by growing each bacterium in 10 mL of NB overnight at 30 °C and 170 rpm. Then, 500 µL of the overnight bacterial suspension was transferred to 200 mL of fresh NB in a 500 mL conical flask and incubated for 52 h (log phase) at 30 °C and 170 rpm. The optical density (OD) of the bacterial cells was adjusted to 0.15 at OD_600_ with NB. For the soil application, the potting soil in each pot was augmented with 10 mL of bacterial suspension and mixed well. For the seed treatment, the cabbage seeds (Hybrid Cabbage Agri-Cross F1) were soaked for 2 h in 10 mL of bacterial suspension at room temperature. The bacterized seeds were shade-dried on sterile filter paper. The fungicide metalaxyl (0.1%) (methyl N-(methoxyacetyl)-N-(2,6-xylyl)-DL-alaninate) at a rate of 10 mL/pot was used as the positive control. After 2 days of treatment with the bacterial strains, the potting soil was inoculated with *P. aphanidermatum.* Next, the 3-day-old PDA culture of *P. aphanidermatum* was cut into 8 pieces equally and 1 piece of agar block containing *P. aphanidermatum* mycelium was added to each pot and mixed well. The cabbage seeds were sown in the treated potting media at a rate of 10 seeds/pot. The cabbage seeds sown in the pots that contained *P. aphanidermatum* only (without antagonist or fungicide) served as the control. The experiment was conducted under controlled conditions at the Agricultural Experiment Station, Sultan Qaboos University, in a glasshouse (27/20 °C day/night, 80% relative humidity, with a photoperiod of 16 h light and 8 h dark) and the plants were irrigated with SDW 2 to 3 times per week. The percentage of the cabbage damping-off incidence was recorded 21 days after sowing by dividing the number of diseased plants by the total number of plants × 100. The percentage of disease reduction was calculated using the following formula: (% damping-off incidence in the control-% damping-off incidence in the treatment)/ % damping-off incidence in the control × 100. The experiment was laid out in a Randomized Block Design. There were 3 replicates for each treatment.

### 4.13. Statistical Analysis

The data were analyzed using Tukey’s test (Minitab 18 software, State College, PA, USA). The data concerning the seed germination percentage and percentage of damping-off disease incidence were subjected to arcsine square root transformation prior to the analysis.

## Figures and Tables

**Table 1:** In vitro inhibition of the mycelial growth of *Pythium aphanidermatum* associated with the damping-off of cabbage by endophytic and rhizospheric bacteria from *Moringa* spp.

Plant Species	Source	Bacterial Isolate	Radial Mycelial Growth (cm)	% Inhibition over Control
*Moringa oleifera*	Root (endophytes)	MOE1	5.7 ± 0.1 fgh	28.7
		MOE2	5.6 ± 0 fgh	30.0
		MOE3	5.8 ± 0 efg	27.5
		MOE4	5.6 ± 0 fgh	30.0
		MOE5	5.7 ± 0 fgh	28.7
	Rhizosphere soil	MOR1	7.3 ± 0.1 b	8.7
		MOR2	7.2 ± 0.1 bcd	10.0
		MOR3	5.5 ± 0.1 gh	31.2
		MOR4	6.8 ± 0.1 d	15.0
		MOR5	6.8 ± 0.2 cd	15.0
		MOR6	7.3 ± 0 cd	8.7
		MOR7	7.1 ± 0 bcd	11.2
		MOR8	5.5 ± 0.1 gh	31.2
		MOR9	7.4 ± 0 b	7.5
		MOR10	7.3 ± 0 b	8.7
		MOR11	7.3 ± 0 b	8.7
		MOR12	7.1 ± 0.1 bcd	11.2
		MOR13	7.3 ± 0 b	8.7
*Moringa peregrina*	Root (endophytes)	MPE1	3.0 ± 0.1 j	62.5
		MPE2	5.8 ± 0 efg	27.5
		MPE3	4.8 ± 0.1 hi	40.0
		MPE4	5.8 ± 0 efg	27.5
		MPE5	5.6 ± 0 fgh	30.0
		MPE6	5.6 ± 0.1 fgh	30.0
		MPE7	6.0 ± 0.1 ef	25.0
	Rhizosphere soil	MPR1	5.7 ± 0.1 fgh	28.7
		MPR2	5.8 ± 0.1 efg	27.5
		MPR3	5.6 ± 0 fgh	30.0
		MPR4	5.6 ± 0 fgh	30.0
		MPR5	5.7 ± 0.1 fgh	28.7
		MPR6	5.6 ± 0 fgh	30.0
		MPR7	5.7 ± 0.1 fgh	28.7
		MPR8	5.6 ± 0.1 fgh	30.0
		MPR9	7.1 ± 0 bcd	11.2
		MPR10	6.7 ± 0.3 d	16.2
		MPR11	6.2 ± 0.1 e	22.5
		MPR12	7.3 ± 0 b	8.7
		MPR13	7.1 ± 0.1 bcd	11.2
		MPR14	7.3 ± 0.1 b	8.7
Control (*P. aphanidermatum* alone)		-	8.0 ± 0 a	-

Values are means ± SE (n = 3). Means in a column followed by the same letters are not significantly different at *p* < 0.05 according to Tukey’s test.

**Table 2:** Identity of the 16S rDNA gene sequences of the endophytic and rhizospheric bacterial isolates from *Moringa* spp. with the nucleotide sequences deposited in the GenBank.

Bacterial Isolate	GenBank Closest Match	Source	% Identity	GenBank Accession Number
MOR3	*Pseudomonas indica*	*Moringa olifera*	100	ON928357
MOR8	*Pseudomonas indica*	*Moringa olifera*	100	ON928355
MPE1	*Bacillus pumilus*	*Moringa peregrina*	98.8	ON908543
MPE3	*Paenibacillus glucanolyticus*	*Moringa peregrina*	100	ON908584

**Table 3:** In vitro inhibition of *P. aphanidermatum* by volatile organic compounds emitted by endophytic and rhizospheric bacterial isolates from *Moringa* spp.

Bacterial Isolate	Diameter Mycelial Growth of *P. aphanidermatum* (cm)	% Inhibition
*Pseudomonas indica* MOR3	5.8 ± 0.6 b	28.4
*Pseudomonas indica* MOR8	7.1 ± 0.1 ab	12.3
*Bacillus pumilus* MPE1	7.1 ± 0.3 ab	12.3
*Paenibacillus glucanolyticus* MPE3	6.3 ± 0.1 ab	22.2
Control	8.1 ± 0.2 a	0

Values are means ± SE (n = 3). Means in a column followed by the same letters are not significantly different at *p* < 0.05 according to Tukey’s test.

**Table 4:** Volatile organic compounds produced by *Pseudomonas indica* MOR3 isolated from *Moringa olifera*.

S. No.	Compound	Retention Time (min)	Peak Area %
1	l-Alanine ethylamide, (S)-	0.019	1.39
2	Pentane, 2-methyl-	0.487	4.15
3	n-Hexane	0.656	1.84
4	Heptane, 3,4,5-trimethyl-	4.006	3.22
5	Heptane, 2,4-dimethyl-	4.196	38.41
6	Heptane, 2,3-dimethyl-	5.148	2.45
7	Octane, 4-methyl-	5.37	17.79
8	3-Ethyl-3-methylheptane	11.362	13.36
9	Nonane, 5-(2-methylpropyl)-	11.653	1.39
10	Nonane, 5-methyl-5-propyl-	12.505	1.14
11	Undecane, 5-methyl-	12.724	6.62
12	Nonane, 4,5-dimethyl-	12.975	1.20
13	Dodecane, 4,6-dimethyl-	17.833	7.03

**Table 5:** Volatile organic compounds produced by *Pseudomonas indica* MOR8 isolated from *Moringa olifera*.

S. No.	Compound	Retention Time (min)	Peak Area%
1	Carbon dioxide	1.253	3.78
2	Propanedioic acid, dihydroxy-	1.405	2.30
3	Acetone	1.49	1.38
4	Unidentified	1.529	1.31
5	Pentane, 2-methyl-	1.729	2.57
6	n-Hexane	1.924	1.86
7	Heptane, 3,4,5-trimethyl-	4.778	3.05
8	Heptane, 2,4-dimethyl-	4.939	31.78
9	2,4-Dimethyl-1-heptene	5.405	0.99
10	Heptane, 2,3-dimethyl-	5.762	2.97
11	Octane, 4-methyl-	5.953	19.71
12	Nonane, 5-butyl-	11.28	1.25
13	3-Ethyl-3-methylheptane	11.495	14.63
14	3,8-Dimethylundecane	11.655	3.77
15	Undecane, 5-methyl-	12.814	7.43
16	Hexadecane	23.346	1.23

**Table 6:** Volatile organic compounds produced by *Bacillus pumilus* MPE1 isolated from *Moringa peregrina*.

S. No.	Compound	Retention Time (min)	Peak Area %
1	Carbamic acid, monoammonium salt	1.296	19.29
2	1,4-Pentadiene	1.597	8.03
3	Carbon disulfide	1.709	2.92
4	Pentane, 2-methyl-	1.789	4.53
5	n-Hexane	1.954	3.77
6	Trichloromethane	2.1	0.54
7	1-Butanol	2.456	1.42
8	Pentane, 2,2,4-trimethyl-	2.686	0.86
9	Heptane	2.826	0.4
10	Acetoin	3.019	1.32
11	Hexane, 2,4-dimethyl-	3.278	0.42
12	Heptane, 3-methyl-	3.959	0.43
13	Heptane, 3,4,5-trimethyl-	4.805	1.86
14	Heptane, 2,4-dimethyl-	4.963	18.03
15	2,4-Dimethyl-1-heptene	5.427	0.47
16	Heptane, 2,3-dimethyl-	5.783	1.49
17	Octane, 4-methyl-	5.971	9.78
18	3-Ethyl-3-methylheptane	11.494	4.02
19	Nonane, 5-(2-methylpropyl)-	12.812	1.53
20	Nonanal	12.9	0.55

**Table 7:** Volatile organic compounds produced by *Paenibacillus glucanolyticus* MPE3 isolated from *Moringa peregrina*.

S. No.	Compound	Retention Time (min)	Peak Area %
1	Carbamic acid, monoammonium salt	1.224	4.67
2	Acetone	1.466	6.56
3	Unidentified	1.535	1.38
4	Pentane, 2-methyl-	1.704	0.91
5	Heptane, 3,4,5-trimethyl-	4.76	1.80
6	Heptane, 2,4-dimethyl-	4.92	20.87
7	Heptane, 2,3-dimethyl-	5.75	2.16
8	Octane, 4-methyl-	5.946	15.49
9	Nonane, 5-butyl-	11.278	1.27
10	Nonane, 5-(2-methylpropyl)-	11.495	14.37
11	3-Ethyl-3-methylheptane	11.654	3.73
12	Nonane, 5-methyl-5-propyl-	12.6	1.45
13	Undecane, 5-methyl-	12.813	7.67
14	5-Methylundecane	12.975	2.74
15	Decane, 3,3,6-trimethyl-	13.172	0.66
16	2-Decanone	14.586	1.74
17	Acenaphthylene, dodecahydro-	15.333	8.15
18	Dodecane, 4,6-dimethyl-	17.839	4.39

**Table 8:** Biofilm formation by endophytic and rhizospheric bacterial isolates from *Moringa* spp.

Bacterial Strains	OD at 600 nm
Pseudomonas indica MOR3	0.2258 ± 0.026 b
Pseudomonas indica MOR8	1.3667 ± 0.146 a
Bacillus pumilus MPE1	0.3834 ± 0.040 b
Paenibacillus glucanolyticus MPE3	0.1382 ± 0.004 b
Control	0.1343 ± 0.016 b

Values are means ± SE of OD_600 nm_ of the stained attached bacterial cells. The values given are the means of ten replications. Means in a column followed by the same letters are not significantly different at *p* < 0.05 according to Tukey’s test.

**Table 9:** Effect of antagonistic endophytic and rhizospheric bacteria from *Moringa* spp. on cabbage growth-promoting ability.

Bacterial Isolate	% Germination *	Root Length (cm) *	Shoot Length (cm) *	Vigor Index *
*Pseudomonas indica* MOR3	93 ± 0	8.6 ± 0.6	4.1 ± 0.1	344 ± 28
*Pseudomonas indica* MOR8	91 ± 5.9	8.5 ± 0.6	3.8 ± 0.4	316 ± 37
*Bacillus pumilus* MPE1	84 ± 5.9	7.4 ± 1.2	3.4 ± 0.1	244 ± 17
*Paenibacillus glucanolyticus* MPE3	80 ± 7.7	6.3 ± 0.2	3.3 ± 0.5	217 ± 45
Control	91 ± 5.9	8.0 ± 0.1	3.0 ± 0.3	217 ± 30

Values are means ± SE (n = 3). * Non-significant.

**Table 10:** Biocontrol potential of endophytic and rhizospheric bacterial isolates from *Moringa* spp. against damping-off of cabbage caused by *Pythium aphanidermatum*.

Treatment	Damping-Off Incidence (%)	% Reduction over Control
Seed treatment with *Pseudomonas indica* MOR3	53.3 ± 8.8 ab	27.3
Seed treatment with *Pseudomonas indica* MOR8	46.7 ± 3.3 ab	36.3
Seed treatment with *Bacillus pumilus* MPE1	40.0 ± 11.5 ab	45.4
Seed treatment with *Paenibacillus glucanolyticus* MPE3	53.3 ± 12.0 ab	27.3
Soil application with *Pseudomonas indica* MOR3	50.0 ± 15.3 ab	31.8
Soil application with *Pseudomonas indica* MOR8	46.7 ± 3.3 ab	36.3
Soil application with *Bacillus pumilus* MPE1	30.0 ± 0 b	59.1
Soil application with *Paenibacillus glucanolyticus* MPE3	70.0 ± 10 ab	4.5
Soil application with metalaxyl	40.0 ± 17.3 ab	45.4
Control (*P. aphanidermatum* only)	73.3 ± 3.3 a	-

Values are means ± SE (n = 3). Means in a column followed by the same letters are not significantly different at *p* < 0.05 according to Tukey’s test.

## Data Availability

All data generated in this study are included in the tables and figures.
